# An update on semantic dementia: genetics, imaging, and pathology

**DOI:** 10.1186/s13195-016-0219-5

**Published:** 2016-12-05

**Authors:** Ramon Landin-Romero, Rachel Tan, John R. Hodges, Fiona Kumfor

**Affiliations:** 1 Neuroscience Research Australia, PO Box 1165, Randwick, Sydney, NSW 2031 Australia; 2 School of Medical Sciences, the University of New South Wales, Sydney, Australia; 3 ARC Centre of Excellence in Cognition and its Disorders, Sydney, Australia

**Keywords:** Semantic-variant primary progressive aphasia, Frontotemporal dementia, Primary progressive aphasia

## Abstract

Progressive and relatively circumscribed loss of semantic knowledge, referred to as semantic dementia (SD) which falls under the broader umbrella of frontotemporal dementia, was officially identified as a clinical syndrome less than 50 years ago. Here, we review recent neuroimaging, pathological, and genetic research in SD. From a neuroimaging perspective, SD is characterised by hallmark asymmetrical atrophy of the anterior temporal pole and anterior fusiform gyrus, which is usually left lateralised. Functional magnetic resonance imaging (fMRI) studies have revealed widespread changes in connectivity, implicating the anterior temporal regions in semantic deficits in SD. Task-related fMRI have also demonstrated the relative preservation of frontal and parietal regions alongside preserved memory performance. In addition, recent longitudinal studies have demonstrated that, with disease progression, atrophy encroaches into the contralateral temporal pole and medial prefrontal cortices, which reflects emerging changes in behaviour and social cognition. Notably, unlike other frontotemporal dementia subtypes, recent research has demonstrated strong clinicopathological concordance in SD, with TDP43 type C as the most common pathological subtype. Moreover, an underlying genetic cause appears to be relatively rare in SD, with the majority of cases having a sporadic form of the disease. The relatively clear diagnosis, clinical course, and pathological homogeneity of SD make this syndrome a promising target for novel disease-modifying interventions. The development of neuroimaging markers of disease progression at the individual level is an important area of research for future studies to address, in order to assist with this endeavour.

## Background

Semantic dementia (SD), a progressive neurodegenerative disorder affecting language, was empirically described only relatively recently. In the early 1970s, the conceptualisation of memory into two distinct systems, an episodic system and a semantic system by Tulving [1], coincided with the report by Warrington [2] of three individuals who presented with visual object agnosia, a profound inability to recognise or identify objects. In light of this new memory system and additional assessment, Warrington recognised that the constellation of symptoms of these patients could be conceptualised as an underlying loss of semantic memory. Since this seminal paper, the syndrome, which is characterised by circumscribed but profound loss of semantic knowledge, has been referred to as SD [3, 4] and, more recently, as semantic-variant primary progressive aphasia (PPA) [5]. Less than 50 years later, our understanding of this striking clinical syndrome has advanced. In this review, we will consider how recent studies in imaging, genetics, and pathology over the last decade have informed our knowledge of SD.

Contemporary consensus criteria for SD require individuals to first meet criteria for PPA; i.e. the most prominent clinical symptom to be in the domain of language, and evidence of subsequent impaired activities of daily living. Then, sub-classification as semantic-variant is based on impaired confrontation naming *and* single-word comprehension, with supportive features including impaired object knowledge, surface dyslexia or dysgraphia, spared repetition, and spared speech production. In a series of 100 cases all of whom underwent longitudinal follow-up, the mean age at presentation was 64.2 years but with a range of 40–79 years [6]. There was a 50% survival of 12.8 years indicating a slower progression than in other forms of frontotemporal dementia [6]. Studies of the prevalence and incidence of SD have been relatively limited; however, a recent epidemiology study estimated the prevalence of frontotemporal dementia at 10.8/100,000, with SD accounting for approximately one-third of these cases [7] in line with previous estimates [8]. Whether this prevalence is similar across countries, however, remains to be examined, as most existing epidemiological data hail from European studies.

## Clinical presentation and cognitive profile

Clinically, patients with SD show a speech profile that is relatively fluent but empty of content, producing a pattern of so-called logorrhoea. Importantly, loss of semantic knowledge is observed irrespective of testing modality [9]. Impaired word comprehension is a mandatory feature and patients demonstrate word alienation in that they are able to repeat words such as “violin” or “caterpillar” but have no idea of their meaning. This deficit gradually progresses from low frequency and less familiar words, such as those mentioned, to more common words. Adlam et al. [10] demonstrated that SD patients are also impaired on non-verbal semantic matching tasks, tests of colour knowledge, sound knowledge, and object-use knowledge, which do not require naming or verbal comprehension even from an early stage of the disease. Such findings have provided evidence that, in SD, symptomatology reflects a profound and progressive loss of conceptual knowledge which is not limited to performance on verbal tasks [11]. There is also accompanying surface dyslexia: patients are unable to correctly pronounce irregular words such as pint which they read to rhyme with hint or flint.

In contrast, recent studies have confirmed that episodic memory is relatively preserved in SD, particularly when tasks with minimal conceptual loading are employed [12, 13]. The intact performance on traditional non-conceptually loaded episodic memory tasks converges with the performance of SD patients on autobiographical memory tasks. Patients typically show relatively preserved recollection of recent autobiographical memory in the context of poorer remote autobiographical memory (known as the reverse temporal gradient or step-function), reflecting increased semanticisation of past events (e.g. [14–16]). This is in stark contrast to the compromised ability of SD patients to project forwards in time to imagine possible future events (e.g. [17]). These deficits in future-oriented thought are attributable to semantic processing impairments, and have led to the advancement of the *semantic scaffolding hypothesis* which proposes that semantic knowledge is required to impart structure and meaning during the process of future simulation [18].

Changes in behaviour and social cognition are increasingly recognised in SD [19]. Clinically, SD patients often show mental rigidity and inflexible behaviour. For example, patients may become obsessive in tasks they engage in (e.g. we have noticed patients spending hours completing jigsaw puzzles), food preferences (usually restricted to specific foods), or daily routines (e.g. clockwatching). In addition, SD patients may have increased apathy and changes in eating behaviour, as well as loss of empathy, impaired emotion perception and emotional memories, and reduced theory of mind capacity [20–24]. Over time, many patients become essentially mute with only a limited repertoire of stereotypic phrases and a complete loss of word comprehension. Changes in emotional capacity as well as increased rigid behaviours are associated with higher carer burden (e.g. [25]), and progressive behavioural changes and/or increasing disability leads to residential care in most cases [6] (Table 1).

**Table 1:** Cognitive profile of semantic dementia at presentation

Impaired	Relatively preserved
Confrontation naming	Episodic memory
Word comprehension	Navigation
Object recognition	Visuospatial ability
Autobiographical memory (reverse temporal gradient)	Attention
Future thinking	Processing speed
Emotion perception and empathy	Phonology and syntax
Theory of mind	Non-verbal problem solving

## Imaging

### Structural imaging

**Fig. 1:**
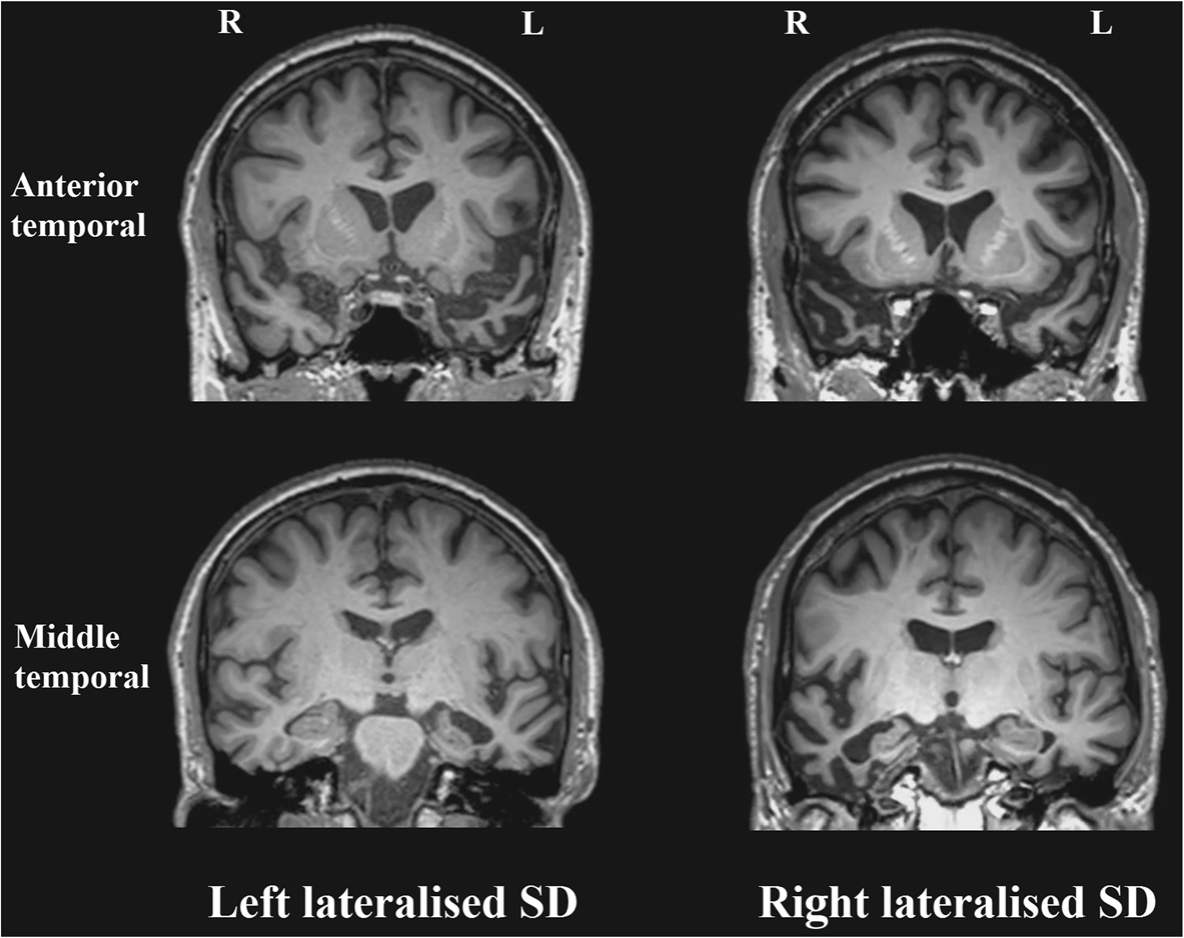
Axial MRI scans showing typical anterior and middle temporal structural abnormalities in early left and right lateralised SD. *L* left, *R* right, *SD* semantic dementia

An extensive body of brain imaging studies have investigated structural and functional brain abnormalities in patients with SD. At presentation, visual inspection of magnetic resonance imaging (MRI) typically reveals hallmark bilateral, but asymmetric atrophy of the anterior temporal lobes, which is usually left lateralised (Fig. 1). With the development of neuroimaging techniques to statistically measure this degeneration, whole-brain structural MRI studies using voxel-based morphometry (VBM) have confirmed grey matter loss, which is relatively localised to the temporal lobe (left-predominant), with some involvement of frontal and limbic regions (e.g. [26]). Specifically, these regions include asymmetric but bilateral involvement of the temporal pole, the fusiform gyrus, middle and inferior temporal gyrus, ventromedial prefrontal cortex, amygdala, hippocampus, and the insula, which have been confirmed by a recent meta-analysis [27]. Importantly, the asymmetric hippocampal involvement is also considered one of the hallmark features of SD. In addition, surface-based imaging studies have demonstrated predominantly left anterior temporal cortical thinning on both the lateral and ventral surfaces of the temporal lobe (e.g. [28, 29]). Debate continues concerning the most critical region, but it appears that bilateral atrophy of the anterior fusiform region is required to generate the syndrome of SD.

More recently, white matter changes in SD have also been mapped using diffusion tensor imaging (DTI) and tractrography analyses. These studies have demonstrated that patients with SD also have reduced white matter integrity in the left temporal lobe, periventricular white matter, corpus callosum, and in white matter tract areas of the fornix, inferior longitudinal fasciculus, and the uncinate fasciculus [30]. Studies using DTI have also shown reduced structural connectivity in frontotemporal pathways in SD, particularly in the uncinate, arcuate, and inferior longitudinal fasciculi [31, 32]. Although a range of DTI metrics have been applied across studies, the areas of abnormality show spatial overlap and are mostly adjacent to regions showing grey matter atrophy [33, 34]. Recently, a tractrography study has uncovered the role of the frontal aslant tract in verbal fluency whilst degeneration of the uncinate fasciculus is uniquely correlated with semantic deficits [35].

**Fig. 2:**
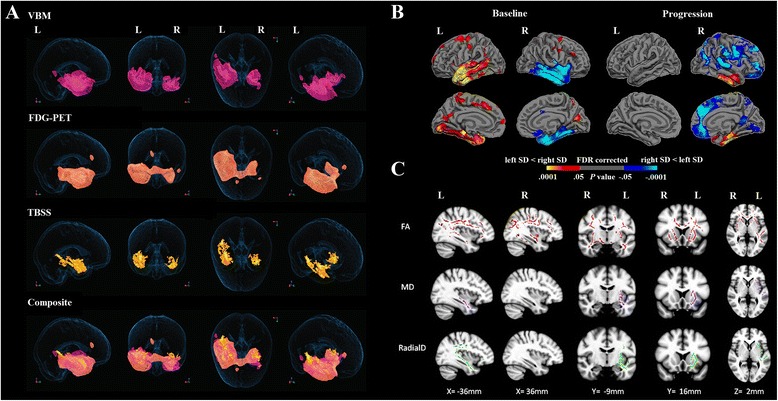
Brain imaging findings in SD at presentation and with disease progression. **a** Cross-sectional multimodal imaging findings in 10 SD patients versus 21 healthy controls: reduced regional grey matter density (*top row*), reduced FDG-PET (*second row*), increased radial diffusivity (*third row*), and composite of multimodal findings (*fourth row*). From Acosta-Carbonero et al. [31] with permission. **b** Baseline and longitudinal changes in cortical thickness in 22 left SD vs 9 right SD patients. From Kumfor et al. [14, 38] with permission. **c** Longitudinal white matter changes from baseline in 11 SD patients. From Lam et al. [40] with permission. *FA* fractional anisotropy, *FDG-PET* fluorodeoxyglucose positron emission tomography, *FDR*, *L* left, *MD* mean diffusivity, *R* right, *RadialD* radial diffusivity, *SD* semantic dementia, *TBSS* tract-based spatial statistics, *VBM* voxel-based morphometry, * ﻿FDR* false discovery rate

Despite the hallmark pattern of atrophy at presentation in this syndrome, how atrophy progresses over time has been relatively poorly understood, in part due to the methodological difficulties in acquiring and analysing longitudinal neuroimaging data. In recent years, however, rapid progress has been made in this area, with an emergence of longitudinal neuroimaging studies tracking progression. These studies have revealed that, in SD, atrophy extends from the anterior temporal lobe into the posterior temporal and/or the inferior frontal lobes with disease progression [36]. Some studies have suggested left greater than right hemisphere atrophy with disease progression [37]. A recent longitudinal imaging study in a larger cohort, however, demonstrated progressive right hemisphere cortical atrophy in SD, despite patients showing left-dominant atrophy at presentation [38]. Longitudinal studies of white matter changes have also revealed involvement of the right hemisphere with disease progression, with focal left lateralized degeneration involving the uncinate and anterior inferior longitudinal fasciculi at baseline, which spreads to the right hemisphere with disease progression [39, 40]. In summary, although the cortical atrophy is relatively localised in the left hemisphere early in the disease, progressive grey and white matter involvement of the frontal and contralateral temporal lobe is observed as the disease progresses (Fig. 2).

### Molecular imaging

In addition to MRI, 2-(Fluorine-18)fluoro-2-deoxy-d-glucose (^18^F-FDG) positron emission tomography (PET) (FDG-PET) [41] has been used to visualize cerebral glucose metabolism, a measure which increases with regional synaptic activity and decreases with synaptic dysfunction or neural degeneration. As such, FDG-PET is a functional imaging marker useful for early diagnosis of dementia, although evidence regarding its utility for differential diagnosis in PPA syndromes is limited [42]. In SD, unsurprisingly, left dominant cerebral glucose metabolism is reduced in the temporal lobes [3], especially the left temporal pole [43] and the hippocampus [36]. These metabolic reductions correspond to observed regional grey matter atrophy patterns [44]. Longitudinal FDG-PET studies have also reported right lateralised reduction in glucose metabolism in the temporal lobes with disease progression, which is associated with impaired cognitive performance [45].

### Functional imaging

Recent studies have also begun to investigate functional brain changes in SD. Functional MRI (fMRI) measures brain activity by detecting changes associated with the blood flow and the blood oxygen level-dependent (BOLD) response. fMRI can be performed either at rest (i.e. resting-state fMRI) or during performance of specific tasks to examine baseline brain activity or activation concomitant with cognitive performance [46]. Whole-brain and regional functional connectivity can be further derived from resting-state fMRI by measuring the correlation of the time series of BOLD signals across brain regions [47, 48]. Resting-state fMRI studies in SD have generally found reduced functional connectivity in the executive [30] and language [32] networks. Seed-based analyses in SD have described extensive disruptions in functional connectivity between the anterior temporal lobe and a broad range of brain regions across the temporal, frontal, parietal, and occipital lobes [49]. Independent component analysis has indicated that SD is also associated with changes in functional connectivity in the prefrontal cortex bilaterally, the anterior cingulate, and in different components within the default mode, salience, and emotion networks [50]. In summary, these results show that SD patients manifest extensive functional connectivity alterations beyond the core atrophic regions in the anterior temporal lobes and related language networks. It should be noted that while these studies have revealed extensive changes in functional connectivity associated with SD, most have not examined how these changes relate to hallmark cognitive and behavioural symptoms in these patients. As such, future studies addressing the behavioural relevance of the observed brain connectivity changes are warranted.

Imaging studies have recently begun to establish the neural correlates of the clinical and cognitive changes associated with SD. In SD, bilateral (yet asymmetrical left > right) neurodegeneration of the anterior temporal lobes is associated with profound semantic deficits, yet syntax, phonology, and fluency are strikingly spared. A set of recent fMRI studies sought to differentiate components of language processing in SD. These studies have described both task-positive and task-negative changes in the language network in SD patients compared to controls showing: i) decreased activation in the fusiform and superior temporal gyrus [51, 52]; ii) increased activation in the intraparietal sulcus [51], the inferior frontal gyrus [52], and the left superior temporal gyrus [53]; and iii) failure of deactivation in the anterior temporal lobe [54]. These results further emphasise the suggested role of the anterior temporal lobes in the combination of both low-level perceptual processing and higher-level integration of semantic processing. Taken together, these findings suggest that spared syntactic processing in SD depends on preserved functionality of left frontal, parietal and, to lesser degree, posterior temporal regions. The relative integrity of regions beyond the medial temporal cortices, such as the posterior cingulate and frontal cortices, also seems to sub-serve other cognitive functions such as the preserved episodic memory performance in SD patients [13, 55].

## Right lateralised semantic dementia

A proportion of patients present with right greater than left lateralised atrophy, referred to as right SD, or right temporal variant frontotemporal dementia [56]. These patients often present with profound behavioural changes, which can make distinction from the behavioural variant of frontotemporal dementia challenging [57]. Importantly, increasing evidence has revealed that the extent of behavioural and social cognition changes is related to integrity of the right temporal pole in this syndrome [21, 38]. Patients with right lateralised atrophy also tend to show greater social cognition deficits than patients with left lateralised SD, while a subset present with prosopagnosia as the primary clinical feature [20, 38]. Improved diagnosis of right SD, together with better understanding of features which give rise to the manifestation of this syndrome, will be important for future studies to address.

## Pathology

Volumetric analyses in autopsy tissue have shown that, in addition to the frontal and anterior temporal cortices, significant degeneration in the cingulate cortices, anterior thalamus, and hippocampal head is apparent by the end of the disease course [58]. The regions within the limbic memory circuit (also known as the Papez circuit) that remain intact include the mammillary bodies, hippocampal body and tail, and memory relays between these key regions, a finding that almost certainly accounts for the relative preservation of episodic memory in patients with SD [15, 58]. Interestingly, a selective loss of von Economo neurons has been identified in the anterior cingulate cortices in SD [58, 59] and may account for some of the behavioural deficits that emerge with disease progression [19].

**Fig. 3:**
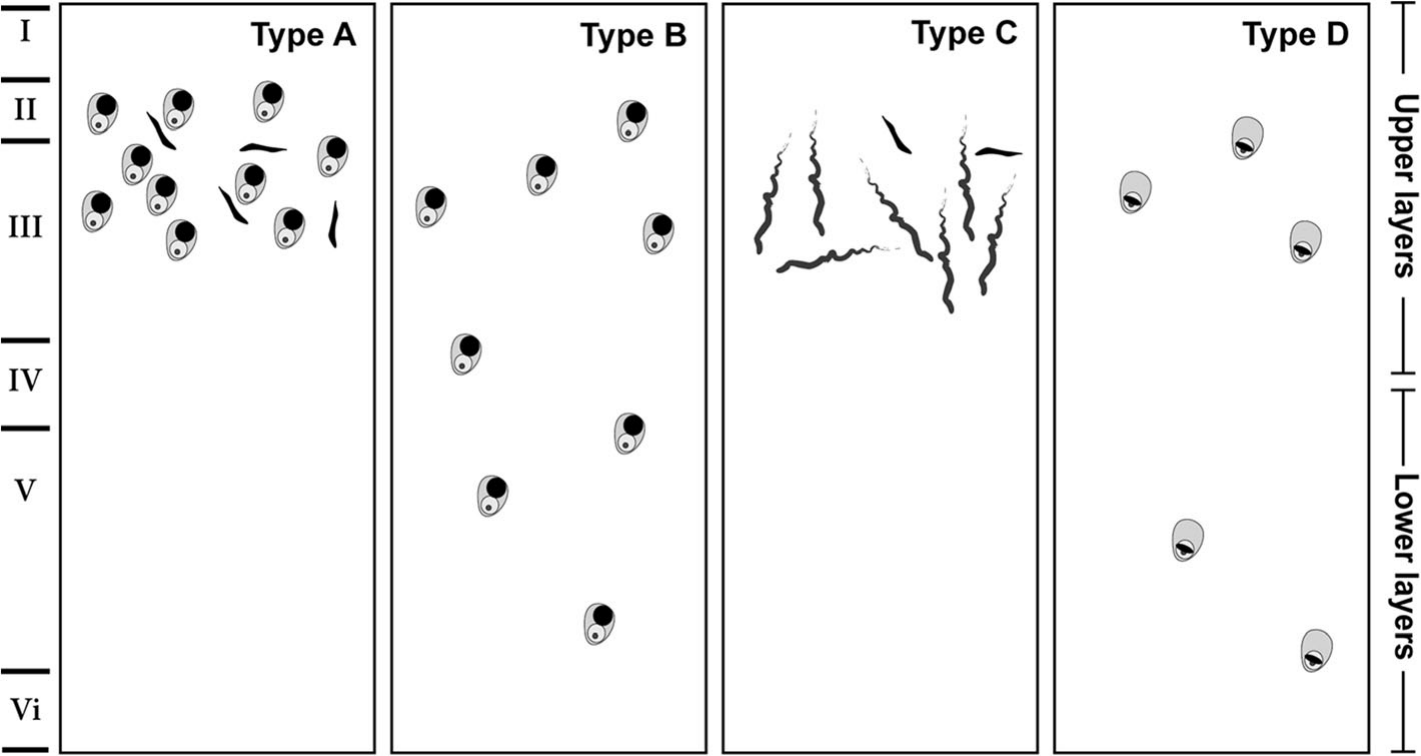
llustration of the FTLD-TDP subtypes. From Tan et al. [63] with permission

At a microscopic level, the main class of pathology identified in patients with SD is frontotemporal lobar degeneration with TAR DNA-binding protein 43 (FTLD-TDP) [6, 60, 61]. Based on the morphology and cortical distribution of the TDP43 lesions, four classes of FTLD-TDP (subtypes A–D) are recognised [62, 63]. In contrast to TDP subtypes A, B, and D, where TDP-43 manifests as neuronal cytoplasmic inclusions with and without short dystrophic neurites and/or intranuclear inclusions, TDP type C is characterised by long dystrophic neurites [62, 63] (see Fig. 3). Notably, clinicopathological studies have consistently found TDP type C to account for the majority of patients with SD. Neuroimaging analyses of TDP subtypes have found TDP type C to be associated with asymmetric anterior temporal lobe atrophy, but this likely reflects the high representation of SD in patients with this pathological subtype [64]. Prominent left anterior temporal thinning is also seen in patients with SD who have FTLD-ubiquitin pathology at autopsy [65].

Nevertheless, FTLD-TDP types A and B, Tau-positive frontotemporal lobar degeneration (FTLD-tau) and Alzheimer’s disease (AD) have been reported in 17% to 32% of patients with SD [6, 64, 66, 67]. Based on the morphology and distribution of the predominant species of tau deposited, FTLD-tau cases are subclassified into 3R (i.e. Pick’s disease) and 4R tauopathies (i.e. corticobasal degeneration, progressive supranuclear palsy and, more recently, globular glial tauopathy) [68, 69]. Almost all patients with SD and underlying FTLD-tau pathology demonstrate the 3R tauopathy Pick’s disease [6, 61, 67], with only one recent report of a patient in which the 4R globular glial tauopathy was identified [70].

Finally, recent research efforts have attempted to identify FTLD pathological subtypes via cerebrospinal fluid (CSF) biomarkers. Increased neurofilament light chain (NfL) levels in the CSF are associated with neuronal and axonal degeneration, and have been reported in patients with neurodegenerative diseases, particularly in patients with probable TDP43 pathology [71–74]. Specifically, increased NfL levels in the CSF have been reported in patients with SD [71, 72]. Although preliminary, these studies indicate that increased NfL levels in the CSF may represent a promising marker of underlying TDP pathology. In summary, emerging clinicopathological research demonstrates that, in the vast majority of cases, TDP43 type C is the most common pathological subtype, rendering SD one of the most pathologically homogenous frontotemporal syndromes.

## Genetics

Unlike other forms of frontotemporal dementia, SD is typically sporadic, and a suggestive family history is identified in around 5% of patients only [6, 75]. In the patients that do have a highly positive family history of dementia, mutation in the progranulin (*GRN*) or expansion of the chromosome 9 open reading frame 72 (*C9ORF72*) gene have been described [76, 77]. Notably, however, these mutations are very rarely found in sporadic SD [78] and the *C9ORF72* expansion has only been described in two patients, both of whom demonstrated co-existing behavioural changes [79, 80].

Interestingly, although a diagnosis of SD in association with a frontotemporal dementia genetic abnormality is rare, semantic impairment appears to develop quite commonly in individuals carrying a mutation in the *GRN* or microtubule associated protein tau (*MAPT*) genes [75, 81]. Given that SD is consistently associated with FTLD-TDP type C pathology, the rarity of mutations identified in this syndrome is consistent with other recognized geno-pathological associations within the frontotemporal dementia spectrum (e.g. *GRN* with FTLD-TDP subtype A; *C9ORF72* with FTLD-TDP subtype B; and *MAPT* with FTLD-tau) [62]. With this in mind, in individuals clinically presenting with SD and with an absence of a strong family history, clinicians can be confident that an underlying genetic abnormality is unlikely.

## Conclusions

As this review reveals, despite a relatively short history much knowledge has been gained about the SD syndrome, particularly over the last decade. Indeed, SD appears to be one of the more straightforward frontotemporal dementia subtypes. It has a clear clinical course, which begins with language features and, with progression, affects behaviour and social cognition; this reflects early and relatively circumscribed neurodegeneration of the anterior temporal pole, which encroaches into medial prefrontal and posterior temporal regions as well as into the contralateral hemisphere with disease progression. Pathologically, it is most commonly associated with TDP43 type C and genetic causes are rare.

In spite of this progress, a number of outstanding questions remain which we hope research over the next decade will address. Clinical phenotype is clearly influenced by the laterality of pathology and associated atrophy in this syndrome, with left lateralised atrophy initially manifesting as loss of semantic knowledge (i.e. anomia) and right lateralised atrophy initially manifesting as loss of person knowledge (i.e. prosopagnosia, knowledge of social norms), giving important insights into the representation of conceptual knowledge across hemispheres [36, 82]. Currently, it remains unclear why only a subset of cases (~30% [56]) present with right lateralised atrophy, with the majority of patients presenting with left lateralised neurodegeneration. Future studies that consider pre-clinical variables (e.g. handedness, occupational history, learning disabilities) with the potential to influence vulnerability of brain hemispheres to disease, may shed light on this issue (e.g. [83]). Indeed, it has been suggested that patients with PPA have a higher rate of pre-existing language disorders than would be expected in the general population, but this has not been investigated specifically in the SD clinical phenotype [83]. In addition, from a management perspective, changes in behaviour, capacity to engage in social situations, and reduced empathy lead to increased burden and stress in carers, which is often greater than in carers of patients with the behavioural variant of frontotemporal dementia [14]. This may reflect inadequate psychoeducation of carers regarding the manifestation of behavioural change in SD patients which is important for clinicians to consider when interacting with family members and carers of SD patients.

Clinically, one of the key issues on the horizon is the development of drugs that target the deposition or clearance of pathology. As these are likely to be specific to pathological subtypes, SD appears to be a promising syndrome for drug developers to target, given the striking clinicopathological concordance. Development of such agents, however, has been hindered by the lack of suitable animal models showing pathological changes that mirror those seen in SD. Knowledge is also lacking about the basic biological processes that underlie the deposition of TDP43 type C in SD. Recent advances in CSF studies may represent a promising avenue to identify FTLD pathological subtypes in vivo. The development of biomarkers such as these (e.g. CSF NfL, neuroimaging, blood biomarkers) are essential for discrimination, prognosis, and prediction of disease progression in SD. Improved understanding of the pathophysiological mechanisms which give rise to SD will also be essential for the development of novel drug interventions. From an imaging perspective, new techniques to measure change in brain integrity and function with disease progression have already started to make some headway. Applications of these techniques at the individual level are likely to be key to track disease progression and potentially measure the efficacy of interventions as these become available.

## Abbreviations

^18^F-FDG: 2-(Fluorine-18)fluoro-2-deoxy-d-glucose
AD: Alzheimer’s disease
BOLD: Blood oxygen level-dependent
CSF: Cerebrospinal fluid
DTI: Diffusion tensor imaging
fMRI: Functional magnetic resonance imaging
FTLD: Frontotemporal lobar degeneration
MRI: Magnetic resonance imaging
NfL: Neurofilament light chain
PET: Positron emission tomography
PPA: Primary progressive aphasia
SD: Semantic dementia
TDP: TAR DNA-binding protein 43
VBM: Voxel-based morphometry
